# Impact of a two-dose varicella vaccination strategy and public health measures on varicella incidence in Shengzhou City: An interrupted time series study

**DOI:** 10.1097/MD.0000000000049617

**Published:** 2026-07-03

**Authors:** Yugang Shen, Jiawen Qian, Qianmei Liu, Yufang Xu, Keze Zhou

**Affiliations:** a Department of Immunity, Shengzhou Center for Disease Control and Prevention, Shengzhou, Zhejiang, China.

**Keywords:** incidence, interrupted time series analysis, PHSMs, vaccination coverage, varicella

## Abstract

This study aims to evaluate the impact of a two-dose varicella vaccine immunization strategy and public health and social measures (PHSMs) on varicella incidence in Shengzhou City. In this study, PHSMs are defined as non-pharmaceutical interventions – including social distancing, school closures, and case isolation – implemented during the COVID-19 pandemic. Data on reported varicella cases in Shengzhou City from 2006 to 2022 were collected, and interrupted time series regression models were constructed to assess changes in monthly varicella incidence rates during the periods of the one-dose strategy, the two-dose strategy, and the implementation of PHSMs. Interrupted time series analyses showed a significant immediate reduction in monthly varicella incidence of 0.754 per 100,000 (*P* = .023) following the introduction of the two-dose varicella vaccine strategy. A further immediate reduction of 1.363 per 100,000 (*P* = .001) occurred after the implementation of PHSMs. Regarding long-term trends, monthly varicella incidence declined by 0.009 per 100,000 (*P* = .284) following the two-dose strategy, though this change was not statistically significant. In contrast, a significant increase of 0.041 per 100,000 (*P* = .001) was observed after the implementation of PHSMs. Our findings suggest that while the two-dose strategy led to an immediate decline in varicella incidence, it did not demonstrate a statistically significant impact on the long-term upward trend within the study period. This may reflect the time required to achieve sufficient population immunity, limitations in surveillance sensitivity, or other unmeasured ecological factors. PHSMs provided short-term suppression but were insufficient to alter long-term transmission dynamics. Sustained high coverage with the two-dose schedule remains essential, and continued monitoring is warranted.

## 1. Introduction

Varicella is an acute respiratory infectious disease caused by the varicella-zoster virus (VZV), primarily transmitted via respiratory droplets and direct contact, and is most prevalent among children and adolescents. Typical clinical manifestations include fever and a generalized vesicular rash characterized by crops of macules, papules, and vesicles. In severe cases, complications such as encephalitis and pneumonia may occur, which can be fatal.^[1]^ Varicella is highly contagious, with an attack rate of 61 to 100% among susceptible individuals following exposure.^[2]^ According to the latest Global Burden of Disease study, it accounts for an estimated 140 million infections, over 4 million hospitalizations, and approximately 4200 deaths globally each year.^[3]^ In China, varicella is the third most prevalent vaccine-preventable disease, with the total number of reported cases exceeding 3 million in 2019.^[4]^ However, the true burden of disease is likely substantially underestimated because varicella is not a statutorily notifiable infectious disease. Since 2006, Shengzhou City has operated a 24-hour direct reporting system for varicella, providing a robust and continuous data source for this study.

Immunisation with the live attenuated varicella vaccine (VarV) remains the most cost-effective strategy for the prevention and control of varicella. Evidence from the United States indicates a 71 to 84% reduction in varicella morbidity within 5 years following the introduction of a single-dose vaccination programme.^[5]^ In China, VarV has been available since 1998,^[6]^ and approximately 60% of children have received a single dose.^[7]^ However, clusters of outbreaks and vaccine failures continue to be reported in school settings.^[8]^ Evidence-based research has demonstrated that a two-dose regimen increases overall vaccine efficacy to over 92%, with near-complete protection against severe disease.^[9]^ Building on this evidence, Shengzhou City implemented a strategic shift in July 2014. The first dose of VarV was offered to children aged 12 to 18 months, followed by a second dose at 3 to 4 years, with a minimum interval of 3 months between doses. Vaccination remained voluntary and self-funded. Since 2017, achieving a varicella vaccination coverage of 70% has been incorporated as a performance indicator within regional immunization planning assessments.

It is noteworthy that public health and social measures (PHSMs) – such as social distancing, mask-wearing, school closures, and case isolation, widely implemented during the COVID-19 pandemic – can also significantly affect the transmission dynamics of infectious diseases. During 2020 to 2022, these PHSMs unexpectedly contributed to a widespread decline in the incidence of respiratory infections.^[10]^ A study conducted in Xi’an reported a 43.18% decline in varicella incidence in 2020 compared with 2019,^[11]^ underscoring the synergistic impact of PHSMs on disease prevention and control.

Although previous studies have shown that a two-dose varicella vaccination schedule is efficacious in controlled settings and serological surveys,^[12–14]^ evidence for its long-term population-level effectiveness remains limited when the schedule is implemented as a public health intervention. This uncertainty is particularly pronounced in urban settings, where population mobility may weaken sustained protection. Moreover, studies have shown that PHSMs can reduce varicella transmission in the short term.^[11]^ However, their long-term interaction with vaccination strategies remains poorly understood. To address this gap, we used an interrupted time-series analysis (ITS) of a 17-year surveillance dataset from Shengzhou City. We defined two sequential intervention points: the introduction of a two-dose varicella vaccination strategy in July 2014 and the implementation of PHSMs in January 2020. We then quantified the short- and long-term effects of each intervention on monthly varicella incidence. Consequently, our study offers new insights into the real-world effectiveness of voluntary two-dose varicella vaccination programmes. It also clarifies the short- and long-term effects of public health emergency measures during the pandemic, without changes to vaccination strategies. These findings may inform future immunization policy in comparable settings.

Shengzhou City was selected as the study site for several reasons. First, it has operated a robust and uninterrupted varicella surveillance system since 2006, yielding a rare longitudinal dataset well suited to ITS analysis. Second, the transition from a one-dose to a two-dose varicella vaccination strategy in July 2014 created a natural experiment through which the effects of this policy change could be evaluated. In addition, as a medium-sized urban center in Zhejiang Province with well-developed transport infrastructure, Shengzhou is broadly representative of typical Chinese cities in terms of population density and mobility patterns that may shape infectious-disease transmission. Finally, the city’s strict implementation of PHSMs during the COVID-19 pandemic provided an opportunity to examine the combined effects of vaccination and non-pharmaceutical interventions on varicella incidence.

## 2. Methods

### 2.1. Study design

Shengzhou is a county-level city located in Zhejiang Province, eastern China, encompassing an area of 1784 square kilometers and home to approximately 690,000 residents. Geographically, Shengzhou borders Hangzhou, Ningbo, and Taizhou, serving as a significant transportation hub. This well-developed transport network facilitates rapid connectivity, which can play a critical role in the swift transmission of infectious diseases.

Three distinct periods were defined according to the implementation of different VarV immunization strategies and PHSMs. The period from January 2006 to June 2014 corresponded to the single-dose strategy. From July 2014 to December 2019, the two-dose strategy was in place. Lastly, the period from January 2020 to December 2022 marked the implementation of PHSMs aimed at curbing the spread of COVID-19.

### 2.2. Data sources

The varicella case data used in this study were extracted from the China Information System for Disease Control and Prevention, a national notifiable disease reporting platform managed by the Chinese Center for Disease Control and Prevention. The time series was constructed using the date of case reporting, as all diagnosed cases are required to be reported within 24 hours through Shengzhou’s direct reporting system established in 2006. These data are not publicly available due to privacy and regulatory restrictions under Chinese public health laws. Ethical approval and data usage permissions were obtained from the institutional review board of the Shengzhou CDC. (Approval No.: 2025009). Given that this study involved secondary analysis of anonymized surveillance data collected as part of routine public health practice, and no identifiable private information was accessed or used, the requirement for individual informed consent was waived by the ethics committee in accordance with Chinese regulations on public health surveillance. Population statistics were sourced from the publicly available Shengzhou Statistical Yearbook published annually by the Shengzhou Municipal Bureau of Statistics.

In this study, VarV vaccination coverage was calculated by birth cohort using data from the Zhejiang Provincial Immunisation Information System, which has maintained demographic and vaccination records for resident children under 15 years of age since 2006. In July 2014, Shengzhou City implemented a two-dose strategy, under which a second dose of varicella vaccine was administered to children aged 3 to 4 years. Consequently, children born before 2011 predominantly received a single-dose immunization regimen (birth cohort 2006–2010), whereas those born from 2011 onwards received a two-dose regimen (birth cohort 2011–2021).

### 2.3. Data accuracy and quality control

The information system of the Chinese Centre for Disease Control and Prevention maintains data quality through a multi-tiered verification process. Infection control physicians at reporting institutions first review all reported cases. Local CDC staff then verify these reports.

Although varicella is not a nationally notifiable infectious disease in China, Shengzhou City has included it in its public health emergency response plan since 2006. This inclusion has improved reporting consistency and strengthened awareness of prevention and control within healthcare institutions. Varicella surveillance in Shengzhou relies primarily on clinical diagnosis and therefore constitutes a passive surveillance system. Since 2010, however, the Shengzhou Municipal Health Bureau has required all healthcare institutions to report varicella cases within 24 hours. This mandate applies to both public and private providers and has standardized reporting procedures while improving the timeliness and completeness of surveillance data. Throughout the study period, clinicians applied diagnostic criteria consistent with the National Guidelines for Varicella Diagnosis. Laboratory confirmation by PCR was not routinely performed. Because the case definition remained unchanged, this practice is unlikely to have affected trend analysis.

### 2.4. Assessment of seasonality using the *M*-value

To characterize seasonal patterns in varicella incidence, we calculated the *M* value, a commonly used index of monthly concentration. The *M* value is based on monthly incidence proportions, defined as the number of reported cases in each month divided by the annual total. It therefore describes the seasonal distribution of incidence rather than absolute case numbers. This method draws on circular statistics and represents the incidence in each of the 12 months as vectors arranged around a circle. The resultant vector length captures the degree of temporal clustering in disease occurrence. Let ri (*i* = 1, 2...,12) denote the proportion of cases occurring in month i relative to the annual total. We assigned each month an azimuth angle. $\theta_{i}=30^{\circ }\times \left(i-1\right).$ The resultant vector of these monthly proportions is decomposed into its horizontal (*R*_*x*_) and vertical (*R*_*y*_) components:


$$\begin{array}{c} R_{x}=\underset{i=1}{\overset{12}{\sum }}\;r_{i}\cos(\theta_{i}),\;R_{y}=\underset{i=1}{\overset{12}{\sum }}\;r_{i}\sin(\theta_{i}), \end{array}$$

where $\theta_{i}=30^{\circ }\times \left(i-1\right)$.

Substituting the exact trigonometric values for each month yields the following computationally efficient expressions:


$$\begin{array}{c} R_{x}=\frac{r_{2}+r_{6}-r_{8}-r_{12}}{2}+\frac{\sqrt{3}}{2}\left(r_{3}+r_{5}-r_{9}-r_{11}\right)+\left(r_{4}-r_{10}\right); \end{array}$$

$$\begin{array}{c} R_{y}=\frac{r_{3}-r_{5}-r_{9}+r_{11}}{2}+\frac{\sqrt{3}}{2}\left(r_{2}-r_{6}-r_{8}+r_{12}\right)+\left(r_{1}-r_{7}\right); \end{array}$$

The *M*-value is then defined as the magnitude of this resultant vector:


$$\begin{array}{c} M=\sqrt{\left(\overset{2}{\underset{x}{R}}+\overset{2}{\underset{y}{R}}\right)}. \end{array}$$

Among these metrics, *M* is termed the concentration index and ranges from 0 to 1. An *M* value of 0 indicates that cases are evenly distributed across months, with no detectable seasonality. By contrast, an *M* value of 1 indicates that all cases occur within a single month. In practice, values of *M* ≥ 0.3 are commonly interpreted as evidence of marked seasonality.^[15]^

We selected this method because it offers a clear geometric interpretation of seasonal clustering, is robust to moderate data fluctuations, and has been widely validated in studies of infectious diseases such as influenza, measles, and varicella. Furthermore, the resulting *M*-value provides a straightforward criterion for determining whether seasonal adjustment is warranted in our subsequent ITS analysis. The *M*-value was employed not to adjust for seasonality in the ITS model, but rather to empirically assess whether varicella incidence in Shengzhou exhibited a seasonal pattern over the study period. This evaluation informed the decision on whether to include seasonal covariates in the regression model. Given that the overall *M*-value was 0.09 (<0.3), indicating non-significant seasonality, and considering the lack of consistent seasonal peaks across all 3 intervention periods, we opted not to incorporate seasonal adjustment terms into the final ITS model. This approach aligns with standard practice in ITS analyses where unnecessary covariates are avoided to maintain model parsimony unless strong seasonal trends are present.

### 2.5. Interrupted time series design

ITSs were conducted to compare changes in the level and trend of varicella incidence before and after the implementation of different immunization strategies and PHSMs. The segmented regression model used in this study follows the standard approach for interrupted time series analysis as described by Bernal et al.^[16]^ The segmented regression models employed were as follows:


$$\begin{array}{c} \begin{array}{cc} Y_{t} & =\beta_{0}+\beta_{1}\times Time+\beta_{2}\times Intervention\;1+\beta_{3}\times Postsope\;1\\ \; & +\beta_{4}\times Intervention2+\beta_{5}\times Postslope2+\varepsilon_{t} \end{array} \end{array}$$

where the dependent variable *Y*_*t*_ represents the monthly incidence of varicella. Time is a continuous variable coded as the number of months elapsed since the start of the study period (January 2006). Given that the study spans 17 years (from January 2006 to December 2022), this corresponds to a total of 204 months, with time = 0 for January 2006, time = 1 for February 2006, ..., up to time = 203 for December 2022. This linear coding allows the model to estimate both the baseline trend (*β*_1_) and changes in trend following each intervention point, which is standard practice in segmented regression for interrupted time series analysis.^[16]^ Intervention1 was defined as a binary indicator for the two-dose varicella vaccination strategy (0 before July 2014 and 1 thereafter), and Intervention2 as a binary indicator for the implementation of PHSMs (0 before January 2020 and 1 thereafter). Postslope was specified as a continuous variable capturing the post-intervention trend, taking a value of 0 before intervention onset and increasing in parallel with the time variable thereafter. *β*_0_ represents the varicella incidence rate in January 2006, marking the start of the study; *β*_1_ denotes the pre-intervention slope, reflecting the temporal trend in varicella incidence prior to the intervention; *β*_2_ corresponds to the immediate change in level associated with the introduction of the two-dose strategy; *β*_3_ captures the change in slope following this intervention, indicating its long-term effect; *β*_4_ represents the immediate level change during the period of PHSMs implementation; *β*_5_ reflects the change in slope in the third segment, representing the long-term impact of PHSMs on monthly varicella incidence; and εₜ denotes the random error term, accounting for variance unexplained by the regression model.

### 2.6. Sensitivity analyses

To assess the robustness of our findings, we performed two sensitivity analyses: alternative model specifications with intervention breakpoints shifted by ±1 and ±2 months to evaluate timing sensitivity; and stratified ITS models by age group (0–4, 5–9, and 10–14 years) to examine whether the effects of the two-dose vaccination strategy and PHSMs differed across populations with varying exposure to each intervention. Consistency of coefficient signs and statistical significance across these models was used to evaluate robustness.

### 2.7. Statistical analysis

Microsoft Excel 2019 was utilized to compile the varicella incidence database and calculate M-values. Statistical analyses were conducted using R version 4.4.2. ITSs necessitated that the time series data be free of autocorrelation; therefore, the Breusch–Godfrey test was employed to assess autocorrelation within the series. Time series without autocorrelation were analyzed using the ordinary least squares method; those exhibiting first-order autocorrelation were analyzed via the generalized least squares approach, implemented using the Prais–Winsten method; while time series with higher-order autocorrelation were addressed using the Newey–West method. Statistical differences in regression coefficients were assessed using two-sided t-tests, with a significance threshold set at *P* < .05.

## 3. Results

### 3.1. Epidemiological characteristics

**Figure 1.**
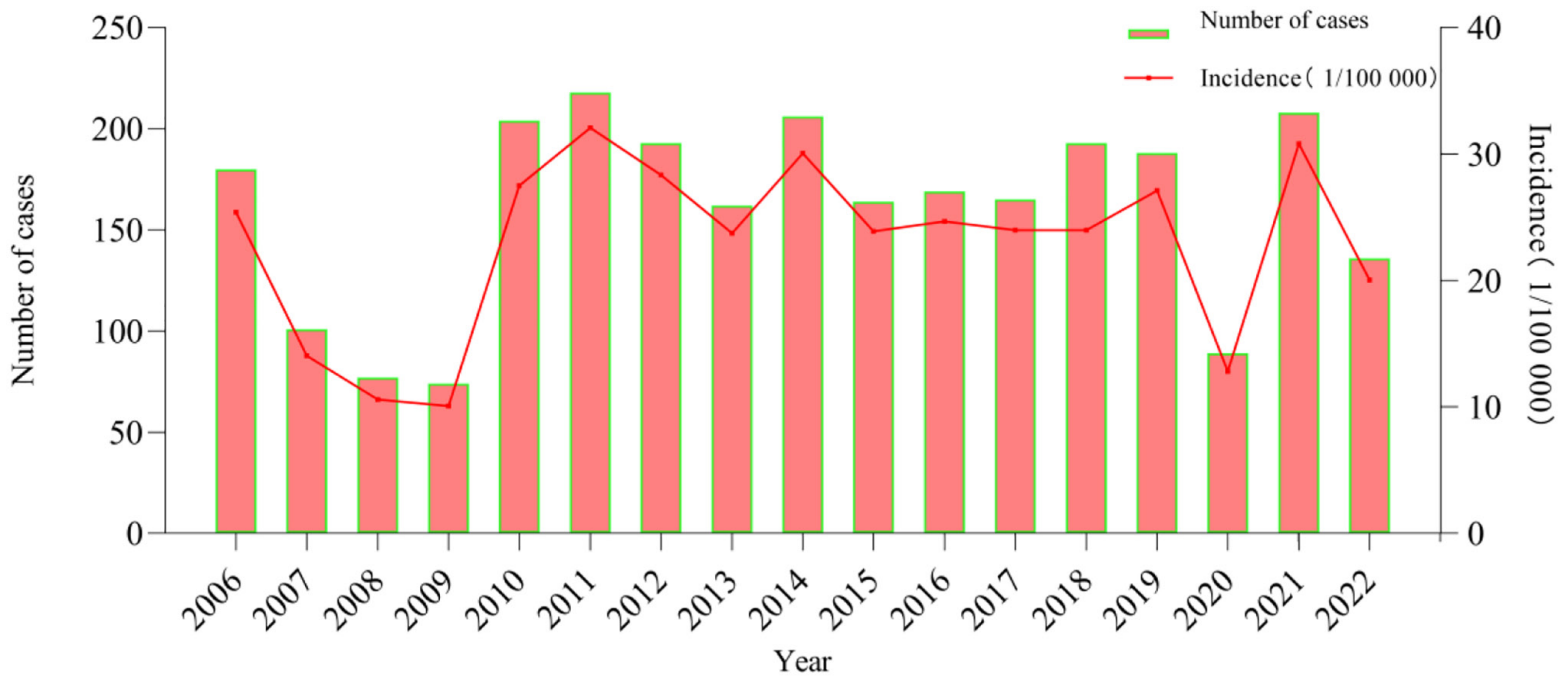
The baseline period represents the single-dose varicella vaccination phase (2006–2014).

A total of 2727 varicella cases were reported in Shengzhou between 2006 and 2022, with an average annual incidence rate of 22.78 per 100,000 population (Fig. 1). The period of single-dose varicella vaccination (January 2006–June 2014) was designated as the baseline against which incidence rates in the two subsequent periods were compared. During the baseline period, the mean annual reported incidence of varicella was 20.87 per 100,000 population. This increased to 27.15 per 100,000 during the two-dose vaccination period (July 2014–December 2019), before declining to 21.13 per 100,000 during the period of PHSMs (January 2020–December 2022) (Fig. 1).

Between 2006 and 2022, the mean reported incidence rates of varicella were 87.76, 135.15, 139.79, and 8.29 per 100,000 population among the age groups 0 to 4, 5 to 9, 10 to 14, and ≥15 years, respectively. Significant differences were observed in mean incidence rates across these age groups (χ^2^ = 7968.865, *P* < .001). The mean reported incidence rates of varicella were 25.19 and 20.72 per 100,000 population for males and females, respectively, with a statistically significant difference between sexes (χ^2^ = 25.758, *P* < .001) (Table 1). Relative to the baseline period, the peak incidence shifted to the 10 to 14-year age group during the two-dose vaccination period (234.01 per 100,000), before declining during the period of PHSMs (116.23 per 100,000).

**Table 1:** Age group and gender distribution of reported varicella incidence rates in Shengzhou, 2006–2022.

Characteristic	Janurary 2006–July 2014		August 2014–December2019		January 2020–December 2022		Total	
	Cases	Incidence (1/100,000)	Cases	Incidence (1/100,000)	Cases	Incidence (1/100,000)	Cases	Incidence (1/100,000)
Age group (yr)								
0–4	275	93.41	89	90.33	26	50.50	390	87.76
5–9	526	160.23	158	107.29	18	41.01	702	135.15
10–14	318	101.34	350	234.01	100	116.23	768	139.79
≥15	209	3.85	369	11.67	289	15.48	867	8.29
Gender								
Male	737	22.64	527	30.14	260	24.86	1524	25.19
Female	591	19.02	439	25.88	173	17.25	1203	20.72
Total	1328	20.87	966	27.15	433	21.13	2727	22.78

Demographic and epidemiological characteristics of reported varicella cases in Shengzhou City, 2006–2022, by age group and sex. Incidence rates are expressed per 100,000 population. The baseline period represents the single-dose varicella vaccination phase (2006–2014).

**Figure 2.**
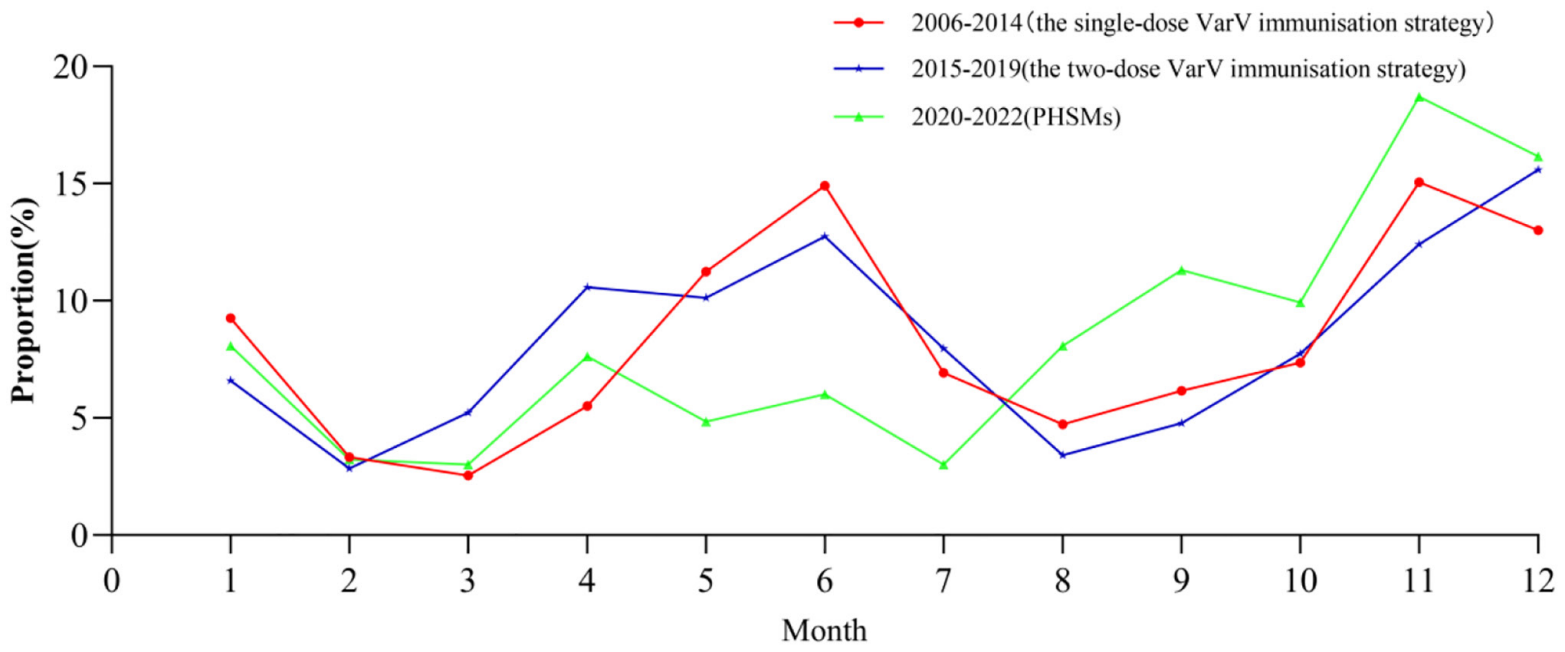
For each period, the number of cases in a given month was divided by the total cases in that period, then averaged across all years within the period.

Between 2006 and 2014, peak varicella incidence occurred in May–June and November–January. During 2015 to 2019, peaks shifted to April–June and November–January, whereas from 2020 to 2022, only a single peak was observed, occurring between November and January (Fig. 2). The overall *M*-value for the average monthly reported incidence of varicella from 2006 to 2022 was 0.09, indicating that varicella incidence was generally non-seasonal. Consequently, seasonal control variables were excluded from the interrupted time series model. The *M*-values for monthly varicella incidence were 0.08 and 0.02 during the periods 2006 to 2014 and 2015 to 2019, respectively, while the 2020 to 2022 period exhibited some seasonality, with an *M*-value of 0.32.

These descriptive patterns characterize the epidemiology of varicella in Shengzhou and underpin key assumptions in our ITS. First, the weak overall seasonality (*M* = 0.09) supported the exclusion of seasonal dummy variables from the regression model, thereby reducing the risk of overfitting. Second, the shift in incidence towards older children during the two-dose period suggests partial vaccine effectiveness that delays but does not prevent infection. This pattern is consistent with the absence of a sustained decline in incidence after 2014. Finally, the abrupt change in seasonality during 2020 to 2022 (*M* = 0.32, unimodal peak) coincided with the implementation of PHSMs and supports the significant immediate reduction in incidence identified by the ITS model.

### 3.2. VarV vaccination status

**Figure 3.**
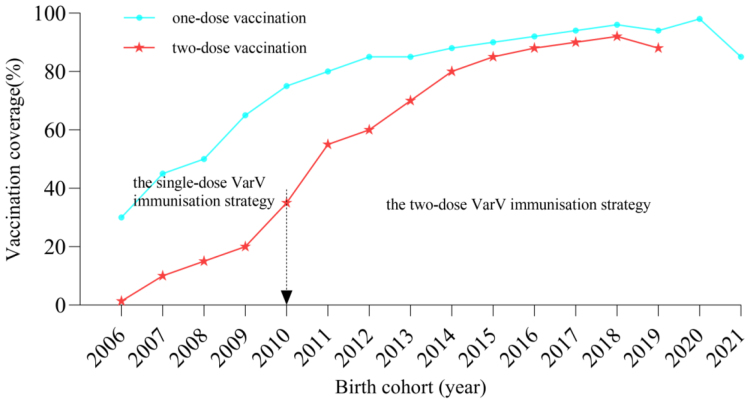
In July 2014, Shengzhou City implemented a two-dose strategy, under which a second dose of varicella vaccine was administered to children aged 3–4 yr. Owing to the low uptake of the second dose during the initial implementation phase, children born before 2011 were classified as belonging to the single-dose immunization period (birth cohort 2006–2010). A small proportion of this cohort nevertheless received a second dose through emergency vaccination during school varicella outbreaks or via vaccination outside Zhejiang Province. Children born from 2011 onwards were classified under the two-dose immunization period (birth cohorts 2011–2021).

Vaccination coverage varied substantially across birth cohorts according to the prevailing immunization policy (Fig. 3; Table 2). Under the single-dose policy (2006–mid-2014), first-dose coverage among children born between 2006 and 2010 ranged from 29.35 to 76.53%, whereas second-dose coverage remained below 36%. Following the introduction of the two-dose schedule in July 2014, uptake of the second dose increased steadily, reaching 53.23% in the 2011 birth cohort, 82.36% in 2015, and peaking at 92.36% in the 2018 cohort. During the period of PHSMs (2020–2022), coverage for both doses remained high (>80%) despite temporary disruptions.

**Table 2:** Varicella vaccination coverage by birth cohort and dose, stratified by immunization policy period.

Policy period	Birth cohorts	Dose 1 coverage (%)	Dose 2 coverage (%)
The single-dose strategy (2006–2014)	2006–2010	29.35–76.53	1.36–35.36
The two-dose strategy (2014–2019)	2011–2015	81.23–90.58	53.23–82.36
PHSMs (2020–2022)	2016–2021	85.26–92.25	83.56–92.36

Varicella vaccine (VarV) coverage by birth cohort and immunization policy period in Shengzhou City, 2006–2022. Coverage is shown for the first and second doses, with children born before 2011 predominantly under the single-dose policy and those born from 2011 onward eligible for the two-dose strategy.

PHSMs = public health and social measures.

### 3.3. Interrupt time series analysis

The Breusch–Godfrey test indicated the presence of autocorrelation in the time series of monthly varicella incidence rates (Lagrange multiplier = 39.668, *P* < .001); therefore, the Newey–West method was employed to adjust the standard errors of the regression parameters. The fitted equation for the interrupted time series regression model of monthly varicella incidence in the overall population was as follows:


$$\begin{array}{c} \begin{array}{cc} & Y_{t}=1.075+0.015\times Time-0.754\times Intervention\;1\\ & -0.009\times Postslope\;1-1.363\times Intervention2\\ & +0.041\times Postslope2+\varepsilon_{t} \end{array} \end{array}$$

**Figure 4.**
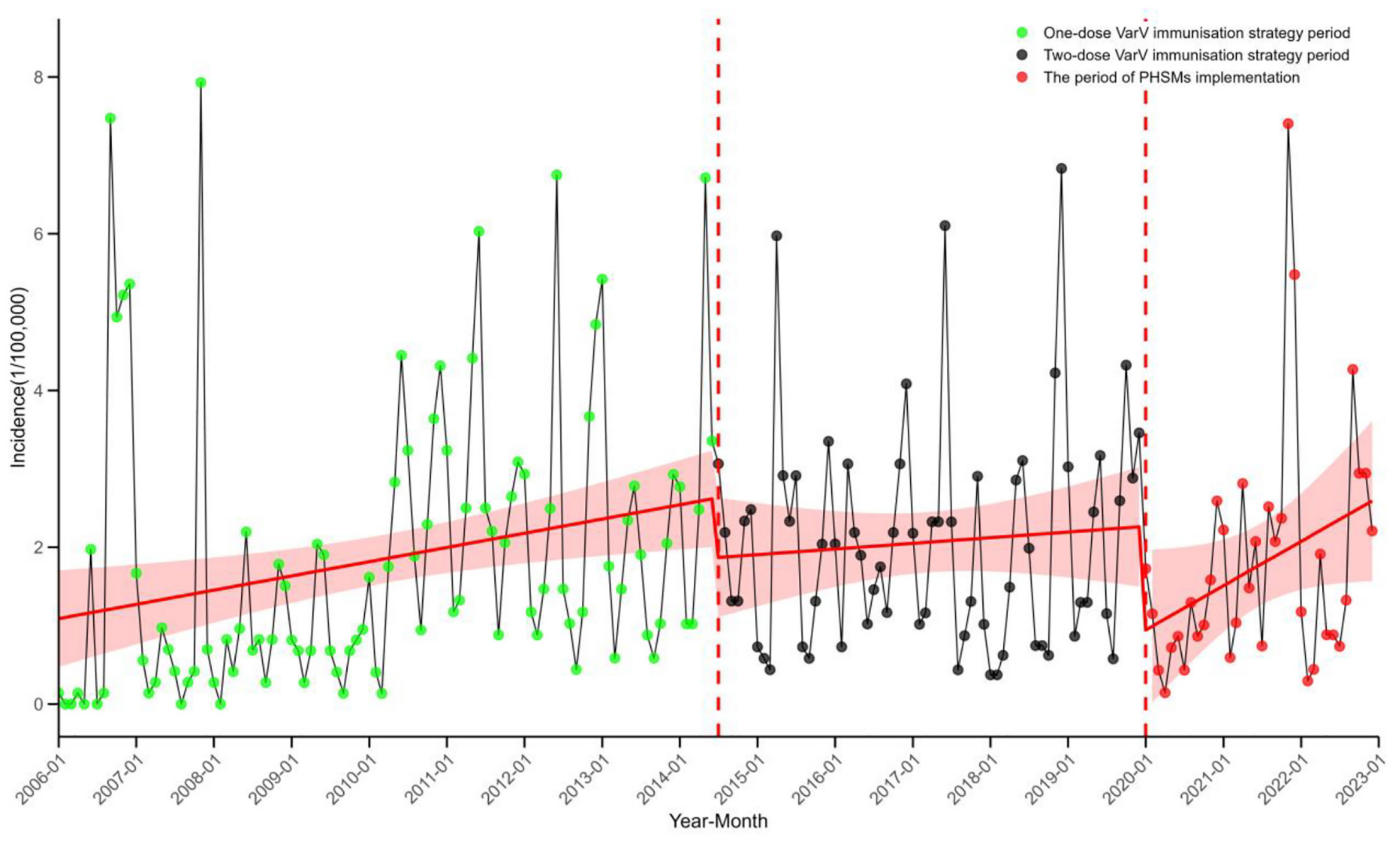
This figure illustrates the segmented regression model used to assess changes in varicella incidence over time. The first intervention point (July 2014) marks the introduction of the two-dose varicella vaccination strategy, and the second intervention point (January 2020) corresponds to the implementation of public health and social measures (PHSMs). Solid lines represent fitted incidence trends within each segment, and vertical dashed lines indicate the timing of interventions.

At the start of the time series, the monthly incidence of varicella was 1.075 per 100,000 population (*β*_0_ = 1.075, 95% CI: 0.190–1.959, *P* = .018), and subsequently increased by an average of 0.015 per 100,000 population per month (*β*_1_ = 0.015, 95% CI: 0.003–0.027, *P* = .016). The first intervention point occurred in July 2014, when the two-dose strategy was implemented. This was associated with an immediate decrease in the monthly varicella incidence rate by 0.754 per 100,000 population (*β*_2_ = −0.754, 95% CI: −1.403 to −0.105, *P* = .023). Following this intervention, the monthly incidence exhibited a long-term increasing trend with a slope of *β*_1_ + *β*_3_ = 0.006 (95% CI: −0.023–0.035). The change in the slope (*β*_3_) was −0.009 and was not statistically significant (*P* = .284). The second intervention point occurred in January 2020, coinciding with the implementation of PHSMs, after which the monthly varicella incidence rate decreased immediately by 1.363 per 100,000 population (*P* = .001). Following this, the incidence exhibited a long-term upward trend, with a significant change in slope (*β*_5_) of 0.041 (*P* = .001). The results of the t-tests for the regression coefficients are presented in Table 3, and the segmented linear regression is illustrated in Figure 4.

**Table 3:** Interrupted time series regression model estimates for monthly varicella incidence rates.

Variable	Estimated value	95% CI	*t*-value	*P*-value
*β*_0_	1.075	(0.190–1.959)	2.40	.018
*β*_1_	0.015	(0.003–0.027)	2.42	.016
*β*_2_	−0.754	(−1.403 to −0.105)	−2.29	.023
*β*_3_	−0.009	(−0.026 to 0.008)	−1.07	.284
*β*_4_	−1.363	(−2.171 to −0.554)	−3.32	.001
*β*_5_	0.041	(0.010–0.072)	2.61	.001

Interrupted time series regression coefficients for monthly varicella incidence per 100,000 population in Shengzhou City, 2006–2022. The model includes intervention points for the two-dose vaccination strategy (July 2014) and public health and social measures (PHSMs, January 2020).

CI = confidence interval.

Sensitivity analyses confirmed the robustness of our primary findings. Excluding the PHSMs period did not substantially alter the estimated effect of the two-dose strategy. Varying the intervention time points by ±2 months yielded consistent results, indicating that our model was not sensitive to minor changes in intervention timing. See Supplementary Table S1, Supplemental Digital Content 1. Subgroup analyses suggested that the reduction in incidence following PHSMs was more pronounced in school-aged children, consistent with the role of school closures and social distancing. See Supplementary Table S2, Supplemental Digital Content 2.

## 4. Discussion

This study examined the dynamic impact of single- and two-dose VarV immunization strategies, together with PHSMs, on varicella incidence in Shengzhou, Zhejiang Province, China. During the implementation of the one-dose strategy in Shengzhou City from January 2006 to June 2014, the average annual reported incidence rate was 20.87 per 100,000 population, marginally lower than the national level reported for the corresponding period (2005–2015).^[17]^ Between the introduction of the two-dose strategy in July 2014 and December 2019, the average annual reported incidence rate in Shengzhou rose to 27.15 per 100,000 population, yet remained substantially lower than the national rate of 55.05 per 100,000 reported for 2016 to 2019.^[18]^ Following the emergence of the COVID-19 epidemic in early 2020 and the implementation of PHSMs by the local government, the average annual incidence rate of varicella in Shengzhou declined to 21.13 per 100,000 population – lower than that reported in Shanghai over the same period.^[19]^

Age-specific analysis showed the highest incidence of varicella among individuals aged 10 to 14 years (139.79 per 100,000), followed by those aged 5 to 9 years (135.15 per 100,000) and 0 to 4 years (87.76 per 100,000). This distribution aligns with national data,^[17,18]^ showing that children aged 5 to 14 years constitute the primary high-incidence group. The incidence rate was marginally higher in males (25.19 per 100,000) than in females (20.72 per 100,000), suggesting a greater risk among males, potentially due to differences in activity patterns and frequency of exposure.^[20]^ Based on the incidence data reported in this study, the overall monthly reported incidence *M* value was 0.09, indicating no clear seasonality. Prior to July 2014, varicella incidence in Shengzhou City exhibited a bimodal distribution, with peaks occurring between May and June and again from November to January annually. This pattern aligns with findings from related studies in China.^[21]^ Between July 2014 and December 2019, although the varicella incidence maintained a bimodal distribution, the timing of the first peak shifted from May–June to April–June, while the second peak remained unchanged. This shift may be attributed to the change in immunization strategies. After 2020, during the implementation of PHSMs, varicella incidence exhibited a single peak (*M* = 0.32), indicating a clear seasonal pattern. This finding is consistent with a study conducted in Anhui, China,^[22]^ suggesting that PHSMs effectively suppress the transmission of varicella.^[23]^

Our findings highlight the importance of China’s varicella vaccination policy. Although previous studies have shown that the two-dose regimen substantially reduces overall incidence,^[12,13]^ the shift in peak infection age observed in this study – from 5 to 9 years to 10 to 14 years – suggests that the current strategy may not fully prevent transmission among older children and adolescents. This pattern is consistent with recent surveillance data from Ningbo, where Pan et al reported increasing proportions of breakthrough varicella cases among school-aged children despite high two-dose coverage (>70%).^[24]^

Although the ITS quantifies the statistical effects of policy interventions, the descriptive data provide essential context for their interpretation. For example, the increase in incidence among children aged 10 to 14 years after 2014, despite high two-dose coverage, suggests waning immunity or incomplete protection. This pattern may explain the absence of a significant long-term decline in the post-intervention slope. Likewise, the suppression of the spring–summer peak during 2020 to 2022 under PHSMs (Fig. 2) visually supports the model-estimated immediate effect (*β*_4_ = −1.363) and strengthens the ecological validity of the ITS findings.

This study identified a rising trend in varicella incidence in Shengzhou City during the one-dose VarV strategy period from 2006 to 2014. These findings suggest that the single-dose regimen did not achieve the intended reduction in varicella incidence.^[25]^ Following the introduction of the two-dose strategy in July 2014, a significant immediate decrease in incidence of 0.015 per 100,000 population was observed (*P* = .016); however, no significant change in the long-term trend was detected (*P* = .284). In Shengzhou City, the full VarV coverage has reached the 80% target recommended by the World Health Organization^[26]^ since 2017, following the inclusion of VarV coverage in the local immunization performance assessment indicators. Nevertheless, the absence of a demonstrable long-term effect of the two-dose strategy may be attributable to cyclical increases in varicella incidence,^[18]^ heightened reporting sensitivity, or the relatively short observation period, which may be insufficient to capture long-term trends. Notably, we observed an age shift in the highest-risk population during the two-dose period compared to the one-dose period, from children aged 5 to 9 to those aged 10 to 14 years. This finding aligns with results from a study in Ningbo,^[24]^ suggesting that the 10 to 14-year age group bears a greater morbidity risk.

The age-related shift phenomenon may also be associated with the insufficient protective efficacy of a single dose of VarV. In Shengzhou City, the second-dose VarV coverage has increased each year since the immunization strategy changed in July 2014. However, most children born between 2006 and 2010 received only a single dose of VarV. Previous studies indicate that, 3 years after a single dose of VarV, only around 50% of children remain seropositive for VZV antibodies.^[14]^ This level of immunity is insufficient to prevent varicella infection. As antibody titers decline over time, children become increasingly susceptible to breakthrough infection when they reach upper primary and secondary school. Several recent domestic studies report similar findings. Monitoring data from Shanghai’s Pudong New Area for 2016 to 2023 show that breakthrough varicella incidence was significantly higher in individuals who received a single dose of varicella vaccine than in those who received two doses.^[27]^ Using seroepidemiological data, Fang et al showed that only 50.23% of individuals aged 13 to 14 years were seropositive for VZV antibodies, highlighting an age-related immunity gap.^[28]^

Real-world studies show that two doses of VarV offer stronger protection against moderate-to-severe varicella than against mild cases.^[29]^ VZV is highly transmissible. Even with substantial vaccination coverage, clusters of breakthrough infection can occur due to suboptimal vaccination schedules or individual variation in immune responses. Consequently, a two-dose strategy alone may be insufficient to interrupt community transmission, particularly in settings without universal free vaccination programs.

During the outbreak of COVID-19 in China in early 2020, the government implemented PHSMs, including social distancing and mask-wearing.^[30]^ These measures were associated with a reduction in the incidence of respiratory infections, such as varicella.^[10]^ This study found that the implementation of PHSMs led to an immediate reduction in the incidence rate by 1.363 per 100,000 population (*P* = .001), demonstrating their prompt effectiveness in interrupting the transmission of respiratory infections such as varicella, consistent with previous studies.^[19,31]^ Long-term trends indicate that varicella incidence increased during the implementation of PHSMs. In 2020, amid local coronavirus transmission, approximately 80% of vaccination clinics in China suspended services for all vaccines except HepB1, BCG, rabies vaccine, and TAT. Such disruptions in vaccination services risked reducing coverage and potentially triggering outbreaks of measles, polio, and other vaccine-preventable diseases.^[32]^ In Shengzhou, vaccination clinic services were suspended in February 2020 and resumed in March. Subsequently, online appointment systems were introduced to limit the number of outpatient vaccinations, which reduced the convenience of preventive vaccination services for children. Data from the Zhejiang Provincial Immunization Information System indicated that the timely VarV vaccination rate among school-aged children in 2020 was only 42.35%. Meanwhile, as epidemic prevention and control measures entered a “normalization” phase and social interactions resumed – particularly with the reopening of schools – varicella incidence rebounded rapidly, contributing to an upward long-term trend despite PHSM implementation. These findings suggest that PHSMs alone are insufficient to alter the long-term morbidity trends of respiratory infectious diseases, highlighting the importance of increasing varicella vaccination coverage as a critical strategy for disease prevention and control.^[29,33]^

This study has several limitations. As varicella is not a notifiable infectious disease under Chinese law, reliance on departmental reporting – modeled after Category C infectious diseases – may not accurately reflect the true incidence.^4^ Additionally, potential confounding factors such as socioeconomic variables and population migration were not fully accounted for. Although official statistics indicate that the proportion of children aged under 15 years has remained stable at approximately 13.79 to 15.56%, unregistered migrant children may still contribute to modest demographic shifts. Furthermore, because monthly varicella vaccination data were unavailable, we could not adjust vaccination coverage for month-to-month variation. We therefore estimated coverage only by birth cohort. This limitation may have led to misclassification of exposure timing and attenuated the expected immediate effect of the two-dose vaccination policy.

Several factors may affect the interpretation of our findings on the long-term impact of the two-dose strategy. First, the program was introduced gradually after July 2014. Full cohort protection, including herd effects, may therefore take several years to emerge. Second, increased awareness and stricter school-based absenteeism reporting after high-profile outbreaks may have improved case ascertainment over time. Third, Shengzhou’s position as a transport hub linking Hangzhou, Ningbo and Taizhou may have increased population mobility after 2014. Such mobility could have introduced susceptible individuals and sustained transmission. These factors complicate the interpretation of incidence trends. Although the ITS model accounts for intervention timing, it cannot fully capture these dynamic contextual changes.

## 5. Conclusion

In summary, implementing the two-dose strategy has shifted the highest-risk age group for varicella. Continued surveillance is needed to monitor the long-term impact of the two-dose strategy on varicella incidence, while also accounting for the potential influence of factors such as climate, vaccine coverage, and population dynamics. Although PHSMs can serve as a useful complementary measure for varicella prevention, their effect on transmission dynamics remains limited. The cornerstone of future varicella control efforts lies in substantially increasing coverage with the two-dose VarV schedule.

## Author Contributions

**Conceptualization:** Yugang Shen, Jiawen Qian, Keze Zhou.

**Investigation:** Jiawen Qian, Qianmei Liu, Yufang Xu.

**Methodology:** Qianmei Liu, Yufang Xu.

**Writing – original draft:** Yugang Shen.

**Writing – review & editing:** Yugang Shen.




