# Supplementary material for: Impact of a two-dose varicella vaccination strategy and public health measures on varicella incidence in Shengzhou City: An interrupted time series study
Source: Medicine (Baltimore). 2026 Jul 3;105(27):e49617. doi: 10.1097/MD.0000000000049617 (PMC13337096; doi:10.1097/MD.0000000000049617)
Supplement: Supplementary file 1 [file medi-105-e49617-s001.docx]

Supplementary Table S1. Robustness of ITS estimates to ±1-2 month shifts in intervention dates for the two-dose varicella vaccination policy and PHSMs implementation.

| Scenario | Intervention 1 Date | β₂ (95% CI) | p-value (β₂) | Intervention 2 Date | β₄ (95% CI) | p-value (β₄) | β₅ (95% CI) | p-value (β5) |
| --- | --- | --- | --- | --- | --- | --- | --- | --- |
| Base model | Jul 2014 | -0.754  (-1.403 to -0.105) | 0.023 | Jan 2020 | -1.363  (-2.171 to -0.554) | 0.001 | 0.041  (0.010 to 0.072) | 0.001 |
| Shift –2 mo | May 2014 | -0.815  (-1.462 to -0.168) | 0.014 | Nov 2019 | -1.437  (-2.240 to -0.634) | <0.001 | 0.039  (0.008 to 0.070) | 0.014 |
| Shift –1 mo | Jun 2014 | -0.792  (-1.435 to -0.149) | 0.016 | Dec 2019 | -1.398  (-2.198 to -0.598) | 0.001 | 0.040  (0.009 to 0.071) | 0.011 |
| Shift +1 mo | Aug 2014 | -0.710  (-1.352 to -0.068) | 0.030 | Feb 2020 | -1.291  (-2.085 to -0.497) | 0.002 | 0.042  (0.011 to 0.073) | 0.008 |
| Shift +2 mo | Sep 2014 | -0.682  (-1.318 to -0.046) | 0.036 | Mar 2020 | -1.325  (-2.110 to -0.540) | 0.001 | 0.043  (0.012 to 0.074) | 0.007 |

Note: All models adjusted for autocorrelation using the Newey–West method, as in the base model.
