# Supplementary material for: Impact of a two-dose varicella vaccination strategy and public health measures on varicella incidence in Shengzhou City: An interrupted time series study
Source: Medicine (Baltimore). 2026 Jul 3;105(27):e49617. doi: 10.1097/MD.0000000000049617 (PMC13337096; doi:10.1097/MD.0000000000049617)
Supplement: Supplementary file 2 [file medi-105-e49617-s002.docx]

Supplementary Table S2. Age-stratified interrupted time series regression results for monthly varicella incidence (per 100,000)

| Variable | 0-4 | | | 5-9 | | | 10-14 | | |
| --- | --- | --- | --- | --- | --- | --- | --- | --- | --- |
|  | Coefficient (95%CI) | t-value | P-value | Coefficient (95%CI) | t-value | P-value | Coefficient  (95%CI) | t-value | P-value |
| β_0_ | 0.821  (0.121 to 1.523) | 2.32 | 0.022 | 1.203  (0.415 to 1.993) | 3.05 | 0.003 | 1.315  (0.522 to 2.116) | 3.26 | 0.001 |
| β1 | 0.018  (0.004 to 0.032) | 2.51 | 0.013 | 0.021  (0.008 to 0.034) | 3.22 | 0.002 | 0.025  (0.011 to 0.039) | 3.54 | <0.001 |
| β2 | -0.512  (-0.892 to -0.132) | -2.65 | 0.009 | -0.423  (-0.710 to -0.162) | -2.15 | 0.032 | -0.124  (-0.410 to 0.162) | -0.85 | 0.397 |
| β3 | -0.015  (-0.038 to 0.008) | -1.28 | 0.203 | -0.008  (-0.031 to 0.015) | -0.68 | 0.498 | 0.006  (-0.018 to 0.030) | 0.50 | 0.618 |
| β_4_ | -0.935  (-1.482 to -0.388) | -3.38 | 0.001 | -1.682  (-2.360 to -1.004) | -4.92 | <0.001 | -1.450  (-2.130 to -0.770) | -4.25 | <0.001 |
| β_5_ | 0.063  (0.026 to 0.100) | 3.41 | 0.001 | 0.055  (0.020 to 0.090) | 3.12 | 0.002 | 0.031  (0.001 to 0.061) | 2.06 | 0.041 |
